# Leagility in the healthcare research: a systematic review

**DOI:** 10.1186/s12913-024-10771-0

**Published:** 2024-03-07

**Authors:** Xueying Li, Ana Lúcia Martins

**Affiliations:** 1 Business Research Unit (BRU-IUL), ISCTE-University Institute of Lisbon, Avenida das Forças Armadas, 1649-026 Lisbon, Portugal; 2 Department of Medical Education, First Affiliated Hospital of Sun Yat-sen University, Guangzhou, 510080 China

**Keywords:** Leagility, Lean and agile, Healthcare, Healthcare supply chain, Systematic review

## Abstract

**Background:**

Expenditure of healthcare services has been growing over the past decades. Lean and agile are two popular paradigms that could potentially contain cost and improve proficiency of the healthcare system. However no systematic review was found on leagilty in the healthcare research. This study aims at synthesizing the extant literature of leagility in the healthcare area to consolidate its potential and identify research gaps for future study in the field.

**Methods:**

A systematic literature review is conducted following the PRISMA checklist approach. Studies were searched in multiple databases. The selection of articles was executed by dual-scanning of two researchers to ensure quality of data and relevance to the topic. Scientific articles published between January 1999 and November 2023 concerning leagile healthcare are analysed using Microsoft Excel and VOSviewer (version 1.6.18).

**Results:**

Out of 270 articles identified from the inclusion and exclusion criteria, 24 were included in the review. A total of 11 target areas were identified in leagility applications in healthcare. Success and limiting factors of leagile healthcare were classified into macro and micro aspects and further categorized into six dimensions: policy, organization, human resources, marketing, operation management and technology. Moreover, four research gaps were revealed and suggestions were provided for future study.

**Conclusion:**

Leagility in the healthcare context is still being in its infancy. Few empirical validation was found in leagile healthcare literature. Further exploration into the application of theory in various sectors under the scope of healthcare is appealed for. Standardization and modularization, leadership support, skillfulness of professionals and staff training are the factors most frequently mentioned for a successful implementation of leagility in the healthcare sector.

**Supplementary Information:**

The online version contains supplementary material available at 10.1186/s12913-024-10771-0.

## Introduction

Over the past few decades, the healthcare industry has been blooming global-wide and so does the expenditure of healthcare services [1]. According to the work of Shrank and his colleagues, the U.S. spends nearly 18% of the gross domestic product (GDP) in healthcare while approximately 30% of the budget may be considered waste [2]. While in China, national expenditure on health has been climbing up from 2016 to 2021, reaching over 10.8 trillion dollars in 2021 [3]. How to contain cost and in the meanwhile maintain high quality health service delivery, has been in the spotlight since the 1980s [1, 4, 5]. Due to the outbreak of COVID-19, economic burden of the disease becomes remarkably high [6] thus such attempt is an increasingly relevant topic.

While the healthcare industry is in pursuit of efficiency, quality and profitability gains, a number of management concepts have proved successful in the manufacturing industry [7]. Two popular paradigms among them are lean and agile [8–12]. Briefly speaking, lean is to reduce waste in order to increase value to customers [13] while agile aims at staying responsive to market demand [14, 15]. However, each single approach has its specificities. In order to achieve greater excellency, it is proposed by scholars to combine lean and agility together as “leagility” to improve performance of the supply chain [10]. Nevertheless, leagility as a process improvement methodology addressing work redesign, it accelerates healthcare’s transition towards digital technology which tremendously expand the capacity of healthcare organizations [16]. Research and applications have been conducted to transfer the lean concept from manufacturing industry to the field of health care [1, 15, 17, 18], but the discussion of leagility strategy in health care settings arose only more recently [1, 4, 7, 19].

The research gap identified concerning leagile healthcare studies is that knowledge is dispersed and no systematic literature review about leagility specifically in the area of healthcare was found. Although there are several articles on leagility in healthcare, an integration of knowledge on the topic is still scarce. To cover the gap, this study aims at synthesizing knowledge on leagile healthcare and discuss its application to find out the important aspects during its implementation in the context of healthcare. Literatures reveal that the theory has potential to improve healthcare delivery service [20–22]. Thus this study is devoted to answer the following questions: 1) how and where can leagility be used in healthcare settings? 2) what are the factors facilitating or limiting a successful implementation of leagility strategy in healthcare?

## Methods

This systematic review was conducted following the Preferred Reporting Items for Systematic Reviews and Meta-Analyses (PRISMA) checklist approach [23, 24].

### Search strategy

Several electronic databases were considered for relevant articles to maximize the identification of relevant articles: B-on, Web of Science, ABI-inform, Scopus, CNKI, Wanfang and Pubmed. CNKI and Wanfang were considered to include Chinese literature. The search strings in titles, key words and abstracts used to trace studies of the field were “lean” AND “agile” AND “healthcare”, “lean” AND “agile” AND “health service”, “leagile” AND “healthcare”, “leagile” AND “health service”, “decoupling” AND “healthcare”. The search syntax is shown in Table 1. No starting date was set for the retrieval of articles. The earliest retrieved article was published in 1999. A list of references published between 1999 up to November 2023 was generated. All selected articles were imported to Mendeley (version 1.19.8).

**Table 1:** Search syntax

Database	syntax
B-on	TX lean AND TX agile AND TX healthcare
	TX lean AND TX agile AND TX health service
	TX leagile AND TX health service
	TX decoupling AND TX healthcare
Web of Science	lean (ABSTRACT) and agile (ABSTRACT) and healthcare (ABSTRACT)
	lean (ABSTRACT) and agile (ABSTRACT) and health service (ABSTRACT)
	leagile (ABSTRACT) and healthcare (ABSTRACT)
	decoupling (ABSTRACT) and healthcare (ABSTRACT)
ABI-inform	lean AND agile AND healthcare
	lean AND agile AND health service
	leagile AND healthcare
	decoupling AND healthcare
Scopus	((TITLE-ABS-KEY(lean) AND TITLE-ABS-KEY(agile) AND TITLE-ABS-KEY(healthcare)))
	((TITLE-ABS-KEY(lean) AND TITLE-ABS-KEY(agile) AND TITLE-ABS-KEY(health service)))
	((TITLE-ABS-KEY(leagile) AND TITLE-ABS-KEY(healthcare)
	((TITLE-ABS-KEY(decoupling) AND TITLE-ABS-KEY(healthcare)
CNKI	Title, Keyword and Abstract: 精益(精确))AND(Title, Keyword and Abstract: 敏捷(精确))AND(Title, Keyword and Abstract: 医疗(精确))
	Title, Keyword and Abstract: 精益(精确))AND(Title, Keyword and Abstract: 敏捷(精确))AND(Title, Keyword and Abstract: 医疗服务(精确))
Wanfang	全部:(精益) and 全部:(敏捷) and 全部:(医疗)
	全部:(精益) and 全部:(敏捷) and 全部:(医疗服务)
Pubmed	((lean) AND (agile)) AND (healthcare)
	((lean) AND (agile)) AND (health service)

### Inclusion and exclusion criteria

**Fig. 1:**
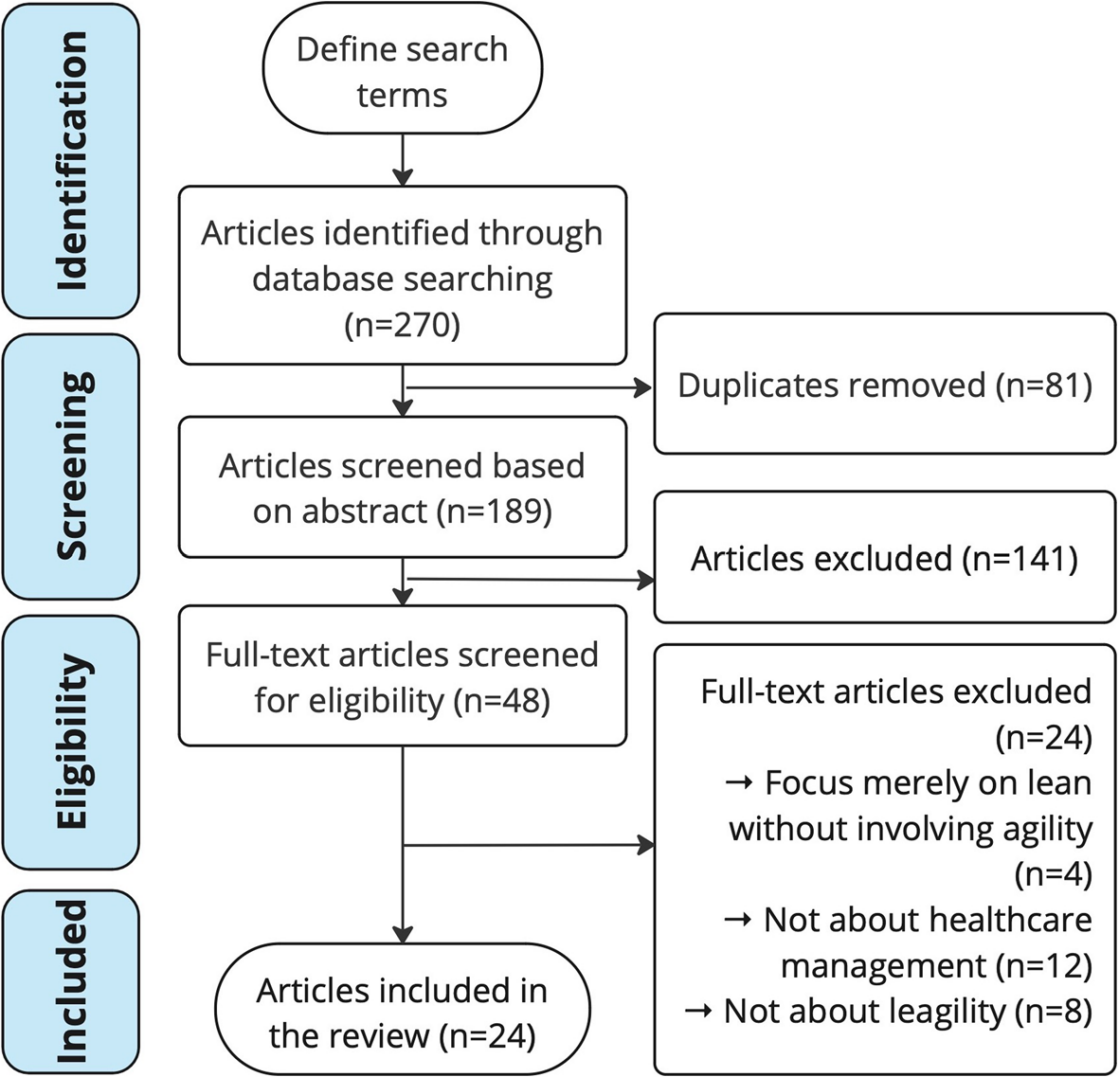
PRISMA flowchart of research selection process

We included journal articles in English and Chinese that relate to leagile healthcare, in other words, decoupling point theory in the healthcare industry. In the screening stage, duplicates and articles that were not relevant to leagile healthcare were removed. Then the remaining studies were analysed for eligibility. In this phase, articles that focus merely on lean without involving agility, that were not about healthcare management, not on leagility or do not satisfy the quality appraisal were excluded. Articles not written in Chinese or English were also ruled out in the study. The flow diagram of screening and selection process is shown in Fig. 1.

### Study selection, data extraction and synthesis

The search strategy was discussed between the two researchers until a consensus was reached. Data were scanned and extracted by two researchers, individual and separately. In this dual-scanning, firstly, the two researchers read the titles and abstracts of the retrieved articles. Secondly, different colors were used to mark whether the article should be included or not, independently by the two researchers. The independency in the screening of the articles aimed at reducing possible bias in the analysis. Included articles were marked in green, excluded ones were marked in red while articles that remained uncertain for classification were marked in yellow. Whenever an article was marked in yellow or in different colors by the researchers, the two researchers went to the full text to determine eligibility of the study by discussion. Evidence was pointed out by one researcher, and ask for agreement of the other. If the other does not agree, more details was provided to support the different opinion. This iterative process was repeated until both sides come to the same decision.

The results of the study consist of descriptive analysis and in-depth analysis. Descriptive data were synthesized according to the year of publication, country or region of the study, journals and their rankings and collaboration of authors among different studies. In-depth analysis includes different perception of leagility in healthcare, methods adopted in the studies, target areas and application of leagile healthcare, applicability of leagility in healthcare sector and what are the success and limiting factors of leagile application in health care. The softwares used in the analysis are Microsoft Excel and VOSviewer (version 1.6.18).

### Quality appraisal of included studies

The quality appraisal of articles was performed by adopting an adjusted assessment checklist for systematic review [25]. The checklist consists of 11 questions related to methods, sampling, quality of data collected and interpretation. In this study, scoring was conducted in this way: articles were scored 1, 0.5, and 0 with a perfect, moderate, or poor quality accordingly.

## Results

### Screening results

A total of 270 articles are identified from searched databases. Eighty-one duplicates are removed and 189 articles remain for further distinction by dual-scanning of two researchers. After analysing the abstracts, 141 articles were ruled out from the study by agreement of both researchers, as they are out of research range of leagility in the healthcare area. The full text of the remaining 48 articles were further screened for eligibility. In this step, 24 articles are excluded as these studies focused only on one aspect of leagile healthcare management but not on the holistic concept. Finally, 24 articles were selected for the systematic review. The PRISMA flow of articles selection is shown in Fig. 1.

### Results of quality appraisal

There are 21 studies recognized as good quality (score of 8 and above), one as medium quality (score between 5.5–8) and one as poor quality (score of 5.5 and below). Generally, all the included studies were fine designed and with clear structure, providing certain insights into the research topic. The report of quality appraisal was attached in Annex.

### Article distribution across reviewed timeframe

**Fig. 2:**
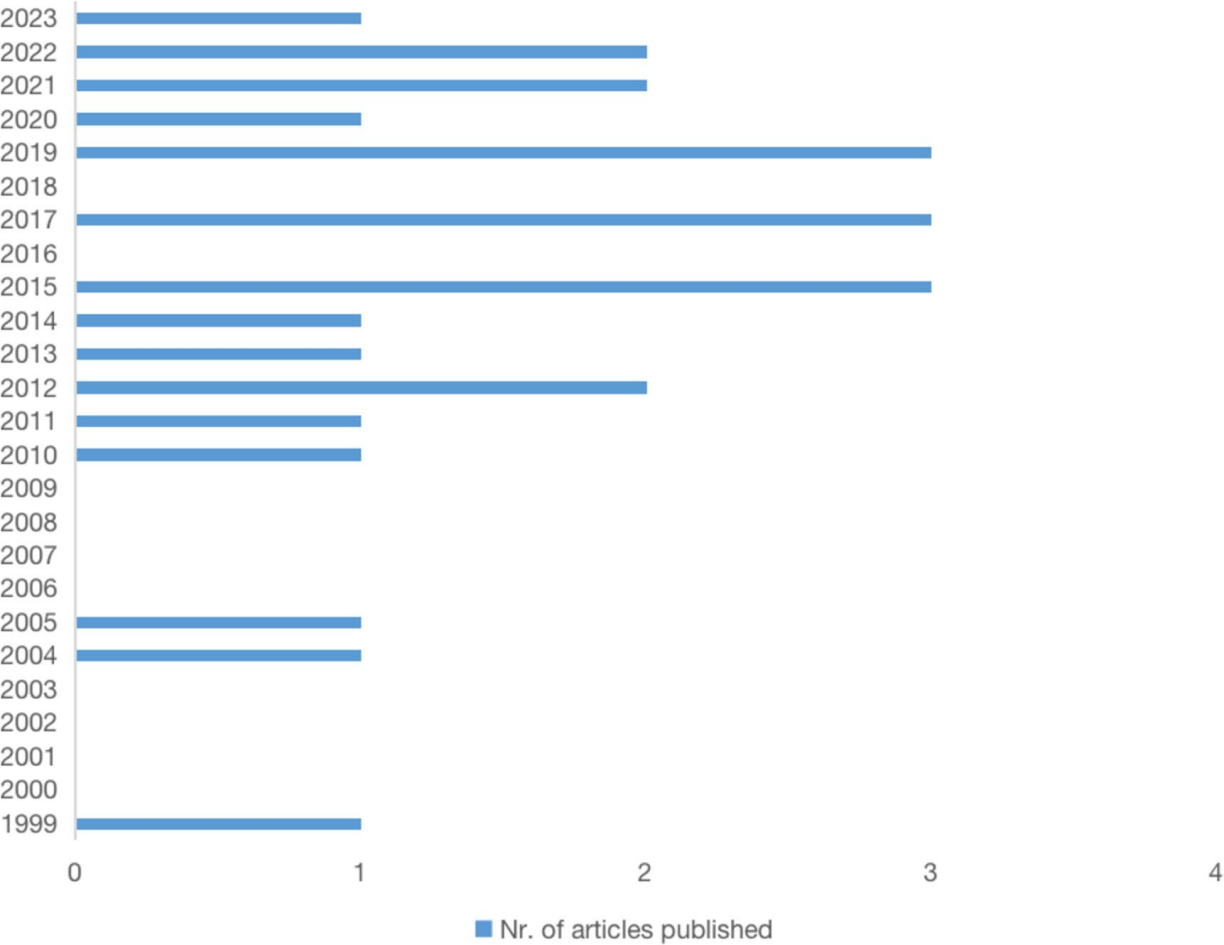
Article distribution across reviewed timeframe (*n* = 24)

A consecutive growth of number of articles focusing on leagility over the reviewed timeframe can be observed in Fig. 2. Articles are published between 1999 and 2023. Among the first decade, ranging from 1999 to 2009, publication on healthcare leagility was very modest, but in the following decade, a boost of publications took place and the growth in number of articles become more stable and continuous. This growth reveals that leagile healthcare is getting more and more attention, which may also result from a recognition of its positive impact on healthcare organizations and settings.

### Geographical distribution

**Fig. 3:**
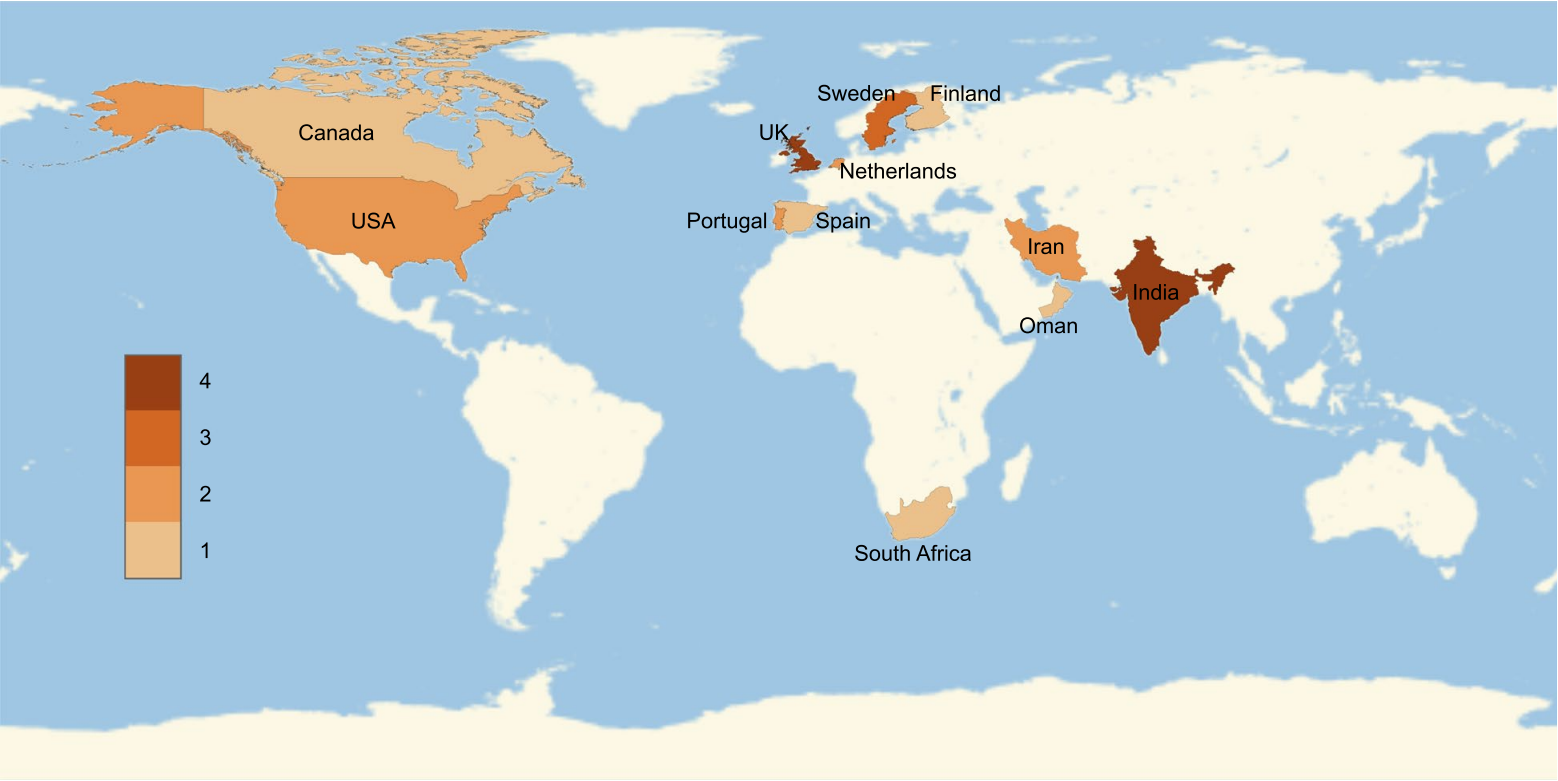
Geographical distribution of selected articles

The published papers report results of research that was carried out in four continents and 15 countries or regions (Fig. 3), showing that the attention given to the topic is not very concentrated. Over half of the research is taken in Europe, while 7 out of 24 studies are embedded in Asia, 3 are conducted in North America and 1 in Africa. The UK and India have more publications on the topic than other countries. This spread of geographical applications show that the impact of using an agility approach in healthcare is not limited to cultural issues. However, research in the topic is yet to be launched in Oceania, Latin America and Africa. It is also observed that leagility in healthcare still remains to be explored on the landscape of China.

With the increase of attention in healthcare in more developed countries, the topic still awaits to be further explored and calls for more in-depth study.

### Journals and rankings

**Fig. 4:**
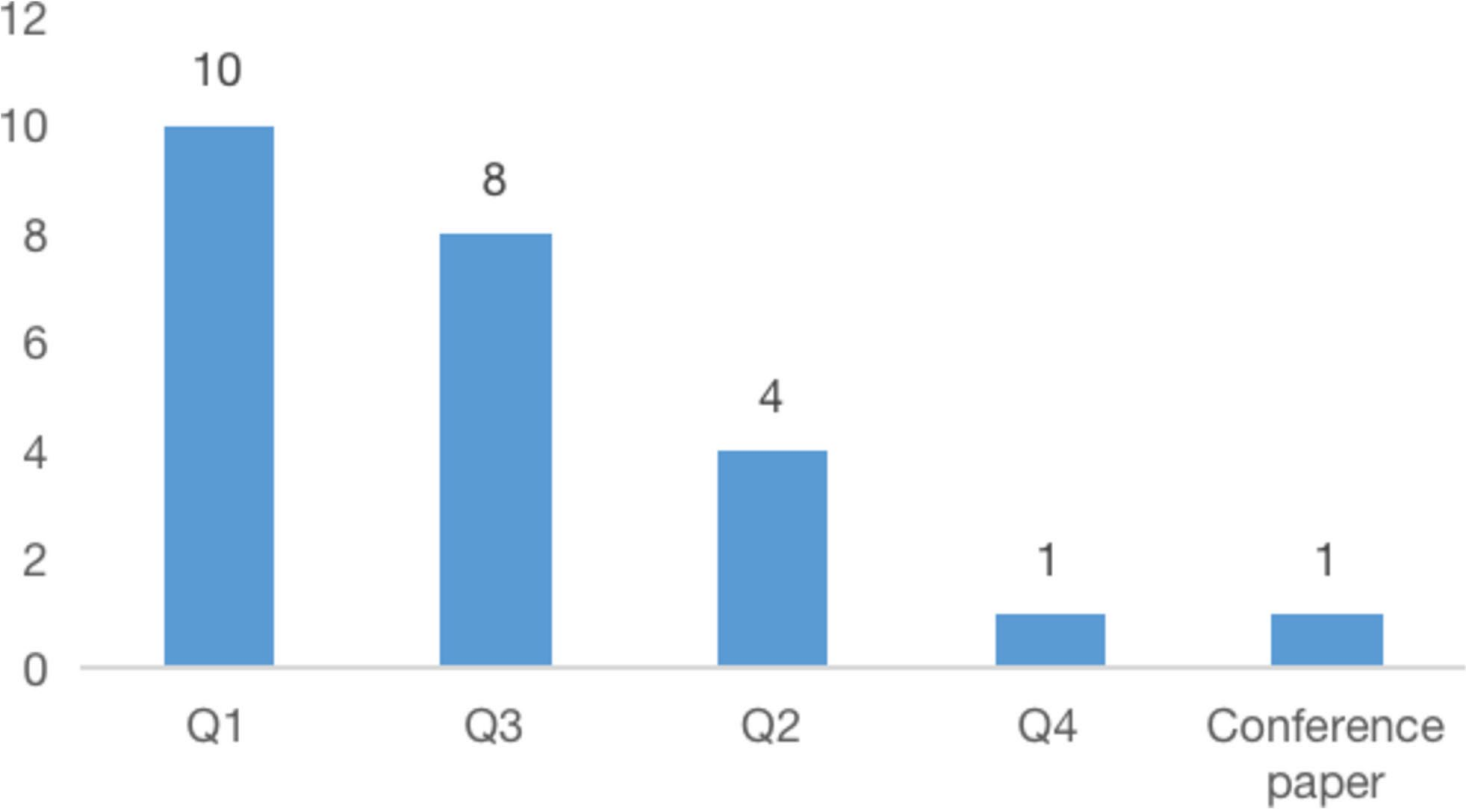
Articles distribution among different journal quartiles

Over half of the articles are published in journals of the first and second quartile, which reflects that studies included in the systematic review are of relatively high quality and recognized impact. The journals that accept more papers concerning leagile healthcare are Supply Chain Management and Production Planning and Control, both of which are ranked in the first quartile. Figure 4 presents the distribution of articles among different journal quartiles.

### Research collaboration in the study of leagility in healthcare

**Fig. 5:**
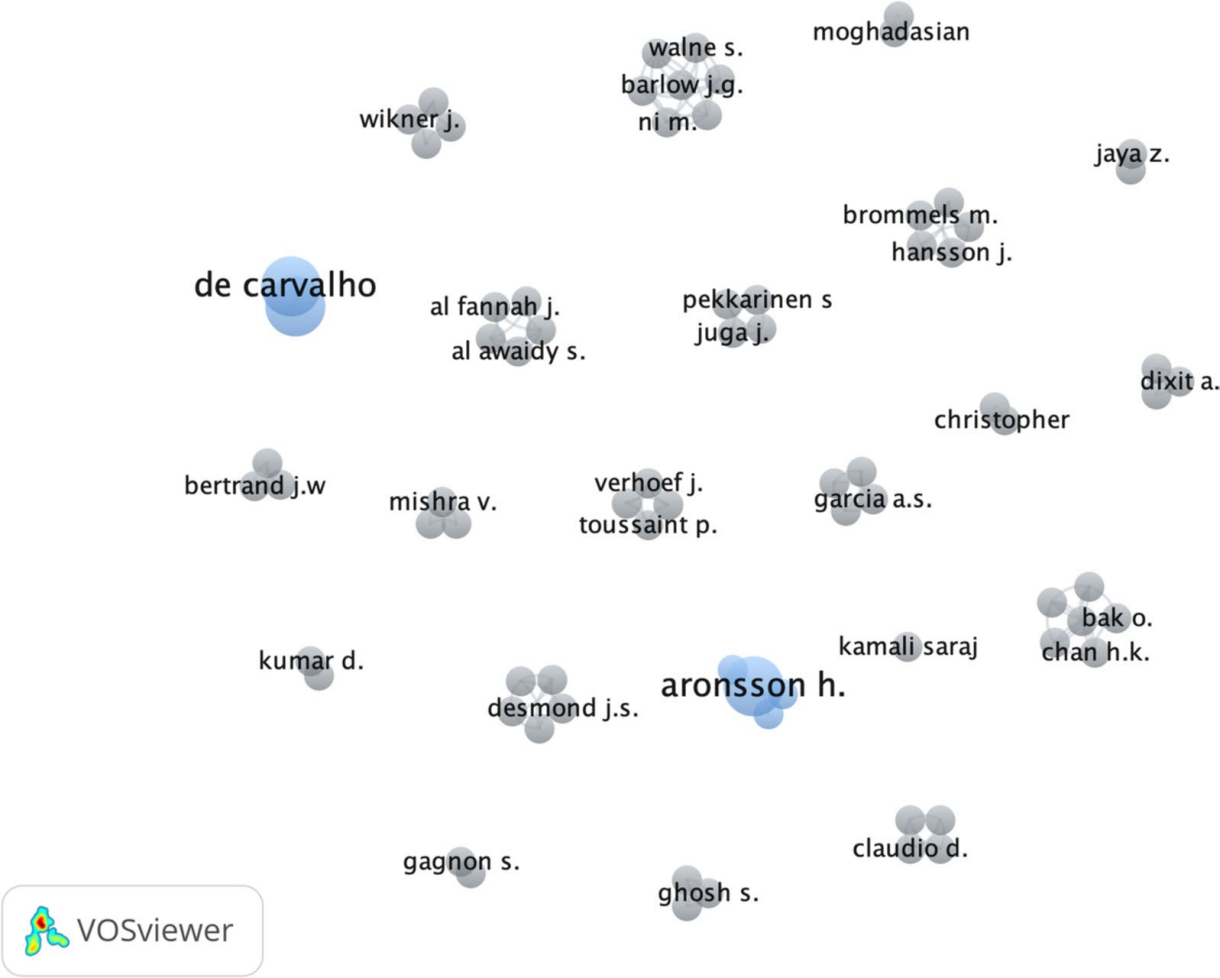
Author collaboration in selected studies

It was previously seen that there is dispersion of geographical application or origin of the publication in the researched topics. This leads to an expectation of a not very high level of collaboration between researchers. This expectation was confirmed. In fact, there are two publications in the pool of articles considered that are by Guimarães and de Carvalho [26, 27] and then only Aronsson [7, 28] published more than one article by working with different partners. Besides these cases, all other publications are by isolated researchers. This limited interaction between research teams is revealed in Fig. 5. This might be the consequence of leagility in the healthcare context still being in its infancy, and eventually with a higher level of recognition of the benefits of the use of a leagile approach in healthcare setting, the collaboration between researchers may increase.

### Perception of leagility in healthcare

The appearance of the concept of leagility can be traced back to as early as 1999 when Naylor and his colleagues proposed the integration of lean and agile paradigms in the total supply chain [10]. Being the combination of two strategies, leagility is also addressed as “hybrid strategy” [7, 20, 26, 29, 30]. In a leagile supply chain, the lean strategy is adopted upstream to reduce waste for maximum productivity and efficiency, while agile strategy serves downstream to satisfy volatile market demand ensuring system responsiveness [1, 7, 28, 29, 31]. The two paradigms are separated by a strategic stocking point called “decoupling point” or “customer order decoupling point” (CODP) where the shift from lean to agile is done at [7, 20, 26, 30, 32, 33]. Sometimes the shift is gradual and the decoupling point can also be a transition point from lean to agile [34].

Nabelsi and Gagnon [35] developed the concept of lean and agile into “patient-oriented, lean and agile”, strengthening the importance of being patient-centered, integrating patient needs within optimized healthcare supply chain. Ni and his colleagues [36] extended the concept into a lean and agile multi-dimensional (LAMP) process, an early health technology assessments framework for evidence generation in commercial decision-making. Furthermore, a lean, agile, resilient and green (LARG) management paradigm has been put forward and attracting increased attention for achieving sustained competitive advantage [37–39]. Claimed to be contested that Leanness is a prerequisite for agility and vice versa [26], it was stated with more certainty later in 2019 that agility is the next step after leaness and agility is best to be achieved when a system is lean [20]. The evolution of the perception of leagility in healthcare in literature is shown in Table 2.

**Table 2:** Perception of leagility in health research

Nr.	Study author	Perception of leagility in healthcare
1	Vries et al. (1999) [40]	Decoupling point in a production flow in healthcare sector can be used as the split between urgent admissions versus elective admission, the split between outpatient care and inpatient care, and the split between diagnostic phase and treatment phase (not used to its fullest potential yet). It is noted that different from manufacturing production, in the third example, the highest variability is upstream in the diagnostic phase rather than downstream in the treatment phase.
2	Toussaint et al. (2004) [41]	The main benefit of applying decoupling in a healthcare organization is that it decreases the interdependency of participants in a joint activity and thus makes the work of each participant more efficient.
3	Towill & Christopher (2005) [42]	A Time-Space Matrix covering the majority of healthcare activities is proposed and the de-coupling point of each pipeline is identified. The de-coupling point intends to lessen the interference between the four healthcare pipelines hence reduces disturbance and sequential falling-off in healthcare supply chain performance.
4	Rahimnia & Moghadasian (2010) [33]	Lean strategy affords markets with predictable demand, low variety and long product life cycle, agility acts best in a volatile environment with high variety and short product life cycle. Combining lean and agility within a healthcare supply chain will help reduce overall lead time and cost of healthcare services. The key challenge in leagile supply chain is to determine the location of the decoupling point, which is used to separate these two paradigms.
5	Aronsson et al. (2011) [7]	The main focus of lean strategy in healthcare is to reduce waste and cost while the one of agile strategy is to ensure fast response to patients, greater flexibility of the system and shorter lead time. It is found that being lean before the decoupling point and being agile after this point is not applicable in a healthcare setting.
6	Saghafian et al. (2012) [22]	Simulation models are used to segment patients into streams for more efficient health care delivery.
7	Guimarães & de Carvalho (2012) [26]	Agile is a post-Lean paradigm leaving to Lean a ″foundational” role. The difference between lean and agile falls mainly on their emphasis on responsiveness to market demand. The combination of lean and agile is called for as individual different care is not addressed when the guidelines shift from mass production to healthcare sector.
8	Guimarães & de Carvalho (2013) [27]	Strategic outsourcing can provide a solution for a flexible, lean and agile healthcare supply chain to deliver better value to the customer.
9	Guven et al. (2014) [32]	Decoupling point (DP) divides the make-to-stock (i.e. push) portion and make-to-order (i.e. pull) portion of a supply chain. There was observed difficulty in locating the DP in a healthcare system thus modelling the process by in-depth study provides a reference to apply the hybrid strategy.
10	Olsson & Aronsson (2015) [28]	Instead of applying lean upstream before the decoupling point and agile downstream after the decoupling point, selecting a strategy for every sub-process based on characteristics such as volume and variety is considered more appropriate in the healthcare sector.
11	Tolf et al. (2015) [29]	Agility prioritizes responsiveness and market sensitiveness to deliver healthcare services based on demand in order to achieve high availability. While Lean prioritize cost optimization by reducing waste to achieve high productivity.
12	Pérez et al. (2015) [21]	Lean improves the operability and efficiency of a clinical laboratory by applying techniques such as zero defects, waste reduction and continuous improvement. An agile methodology enables the flexibility and adaptability to cope with changing needs from physicians to obtain analytical information under specific challenges such as certification and accreditation of the laboratory.
13	Kuupiel et al. (2017) [43]	A lean and agile supply chain management framework helps to improve the accessibility and efficiency of point of care (POC) diagnosis services in low- and middle-income countries.
14	Nabelsi & Gagnon (2017) [35]	A patient-oriented, lean and agile (POLA) approach is proposed for hospitals to integrate healthcare processes and reconcile efficiency imperatives in the supply chain with the adoption of IT technology. Agility helps hospitals respond fastly to patient needs and unexpected risk events, while lean contributes to continuous quality improvement and cost control in the healthcare system.
15	Wikner et al. (2017) [34]	A flow-based decoupling thinking framework combining the back- and front- office distinction and five decision categories for service design is built to introduce the concept of standardization versus customization in a service context. The framework can be applied to healthcare service through patient flow differentiation.
16	Dixit et al. (2019) [1]	Lean operation in healthcare supply chain (HSC) provides high efficiency with low cost while agile operation offers high responsiveness for low service time and quick availability of healthcare services. It is time to move on “leagile operation” by coupling these two for efficient, effective, quick responsive HSC with cheaper cost at the same time.
17	Mishra et al. (2019) [20]	The backend portion of a healthcare service operation such as medicines, equipment, nursing aids and food supplements for chronic disease operates much like a factory, and it could adopt leagility from the manufacturing sector for better management. Besides demand variability, criticality, cost and perishability are characteristics of the product that should be considered when choosing a right strategy for the management of that product.
18	Pohjosenperä (2019) [44]	Modularization and standardization enable value creation in healthcare logistics management.
19	Ni et al. (2020) [36]	A lean and agile multi-dimensional process (LAMP) is developed to generate a minimum set of evidence (lean) relevant to manufacturer needs (agile). The joint execution of lean and agile, or le-agility, produces a process that is highly dynamic and outcome- driven with demonstrable results. Applied to healthcare evidence generation, leagility gives rise to a diffused and iterative approach for healthcare technology design and development.
20	Claudio et al. (2021) [45]	An agile standardized work procedure is proposed for minimizing turnover time and cost for operating room (OR) preparation with varying number of staff available to clean the room.
21	Sen et al. (2021) [30]	Patient Order Decoupling Point (PODP) acts as a buffer to determine the upstream and downstream of the supply chain of healthcare products and services, which results in the synergy between the healthcare service provider and patient through collaboration-co-ordination-cooperation.
22	Fannah et al. (2022) [46]	Agile teams using lean methods streamline the workflow of a hospital under COVID-19 to respond fast to volatile patient demand such as the unexpected inflow of infected patients and elective services resumption. Information system is also adopted for adaptation in order to fulfill healthcare needs during the pandemic.
23	Yadav & Kumar (2022) [31]	Leagility is the integration of lean and agile paradigms. Leanness in a healthcare supply chain increases profits by cutting costs and reducing waste, while agility optimizes profit by supplying exactly what the patient wants. The agile management practices are used where demand is unpredictable, and the lean paradigm is applied where demand is steady.
24	Saraji et al. (2023) [47]	Lean strategy aims at creating a waste-free flow to produce products with less cost and add value to end users. While agility of an organization refers its capacity to produce and deliver unique goods to its customer in a timely and cost-effective way.

### Applied methods

It was found that 19 out of 24 studies adopted a qualitative methodology, as seen in Table 3. Only two articles adopted quantitative methods. Four studies selected mixed methods. The main use of qualitative approaches is also evidence of case applications and research in a topic that is still in its infancy, requiring further attention to be able to expand the knowledge in the topic and its impact in the healthcare care area. It is indicated that the majority of included articles use indirect and secondary data while empirical practice of leagility in the healthcare sector is still scarce.

**Table 3:** Methodology adopted among leagile healthcare research

Methodology	Nr. Of articles	Reference
1. Quantitative	2	
1) Mono-method quantitative	1	Saghafian et al. (2012) [22]
2) Multi-method quantitative	1	Pérez et al. (2015) [21]
2. Qualitative	19	
1) Mono-method qualitative	11	Al Fannah et al. (2022); Aronsson et al. (2011); Dixit et al. (2019); Guimarães & de Carvalho (2013); Kuupiel et al. (2017); Rahimnia & Moghadasian (2010); Sara et al. (2015); Toussaint et al. (2004); Towill & Christopher (2005); Vries et al. (1999); Wikner et al. (2017) [1, 7, 27, 29, 33, 34, 40–43, 46]
2) Multi-method qualitative	7	Guimarães & de Carvalho (2012); Guven et al. (2014); Mishra et al. (2019); Nabelsi & Gagnon (2017); Ni et al. (2020); Olsson & Aronsson (2015); Pohjosenperä et al. (2019) [20, 26, 28, 32, 35, 36, 44]
3. Mixed method	4	
1) Mixed method simple	1	Claudio et al. (2021) [45]
2) Mixed method complex	3	Sen et al. (2021); Yadav & Kumar (2022); Saraji et al. (2023) [30, 31, 47]

### Target area and application

Table 4 explores the area of application of the different considered studies. Almost half of the selected studies on leagile healthcare hold a system-wide or hospital-wide view towards the topic. The second heated application of leagility focus on patient flow. While implementation of leagility in other areas such as pharmacy, medical equipment and clinical laboratory is limited and documented only in recent decade. This show that the recognition of the usability and positive impact of leagility in the healthcare area is becoming more sustained, with overall approaches guiding the application in more detailed areas. Nonetheless, many healthcare areas are still unexplored.

**Table 4:** Target area and application of leagility in health care

Target area	Nr. Of articles	Study Author(s)
Hospital-wide	7	Vries et al. (1999)**;** Towill & Christopher (2005)**;** Guimarães & de Carvalho (2012)**;** Guimarães & de Carvalho (2013)**;** Tolf et al. (2015)**;** Pohjosenperä et al. (2019)**;** Al Fannah et al. (2022) [26, 27, 29, 40, 42, 44, 46]
Patient flow	6	Rahimnia & Moghadasian (2010)**;** Aronsson et al. (2011)**;** Saghafian et al. (2012)**;** Guven et al. (2014)**;** Olsson & Aronsson (2015)**;** Wikner et al. (2017) [7, 22, 28, 32–34]
Overall healthcare system	2	Dixit et al. (2019); Sen et al. (2021) [1, 30]
Pharmacy	3	Nabelsi & Gagnon (2017)**;** Mishra et al. (2019) [20, 35]
Equipments	1	Nabelsi & Gagnon (2017) [35]
Clinical laboratory	1	Pérez et al. (2015) [21]
Healthcare technology	1	Ni et al. (2020) [36]
Point-of-care (POC) diagnostics	1	Kuupiel et al. (2017) [43]
Communication process	1	Toussaint et al. (2004) [41]
Operating room (OR) cleaning	1	Claudio et al. (2021) [45]
Vaccine supply chain	1	Yadav & Kumar (2022) [31]

### Applicability of leagility in the healthcare sector

According to the Global Supply Chain Matrix proposed by Christopher et al., a leagile strategy is best to adopt when a product is of unpredictable demand and long lead time [15]. The applicability of leagility was further analysed by Mishra et al., adding three additional variables: criticality, cost and perishability of the product. It was found that leagile strategy suits best when the product is of relatively low criticality, low cost and highly perishable [20].

In the healthcare area, based on the selected articles and the cases they explore, it is undeniable that the level of demand is unpredictable, mainly if one considers emergency areas [22]. As a service, healthcare capacity is highly perishable, requiring the need to explore the capacity of the resources available and their competencies in the most effective way to, simultaneously, assure the best quality possible in delivery and controlled costs.

### Success and limiting factors of leagile application in health care

The identified success and limiting factors of leagility in the context of healthcare are shown in Table 5. These factors were first divided in macro and micro level and then further categorized into different dimensions according to their nature. For macro aspects, policy assurance in leagility application in healthcare is reported to be important for adequate financial support and political commitment [32, 43]. For micro aspects, factors are classified into five dimensions: organizational, human resources, marketing, operation management and technology. At organizational level, success factors of leagile healthcare that appear more in literature include top-down decision [21, 26, 27], well-established control machanism and monitoring results [40, 47], and understanding of need for better planning and control [21, 28]. But if an organization lacks system-wide strategy and stays only at a tools-and-techniques level, actions are taken to solve local problems only and this limits the maximized impact of leagility implementation [26]. Concerning human resources dimension, professionalism level, staff traning and employers’ engagement pose themselves as more frequently mentioned success ingredients of leagile healthcare. While lack of skillful and experienced professionals hinders the application of leagile strategy [32, 33]. High level of market sensitivity and staying customer focus facilitate the strategy as well [29]. In the aspect of operation management, standardization [7, 27, 34, 45] and modularization of processes are the two elements mostly emphasized in studies to implement leagility in healthcare [7, 33, 34, 42, 44, 45]. What follows is short product life cycles for timely delivery [29, 47] and sufficient use of shared resources [22, 40]. However, out of the difficulty to control and monitor performance, outsourcing might introduce potential risk during implementation of leagility [26]. Moreover, it is worth paying attention to information technology as it has significant impact on an organization’s capability to manage demand and stay responsive as well as flexible in a volatile environment [16, 41, 47].

**Table 5:** Success and limiting factors of leagile application in healthcare

Level	Dimension	Success factors	Limiting factors
Macro	Policy	• Policy ensurement for adequate funding and political commitment [43]	• Imperfect financial regimes [32];
Micro	Organization	• Leadership support/Top-down decision [21, 26, 27]; • Centrally designed financial regimes [32]; • Clearly defined organizational strategy [40]; • Well-established control machanism and monitoring results [40, 47]; • Culture construction at all levels [27]; • Transparent and transient inter-organizational links at all levels [29]; • Coordination among stakeholders [31]; • Strategy and alignment [27]; • Understanding of need for better planning and control [21, 28]; • Assessments of ‘compatibility’ of leagility within clinical context [36]; • Well established control machanism for controling the patient flow and resources [40]; • Management commitment and empowerment [27]; • Innovation ability/skill to satisfy consumer demand [47]	• Lack of system-wide strategy and things stay only at a tools-and-techniques level thus actions are taken to solve local problems only [7, 27, 28]; • Long-term issues not taken into consideration [26]
	Human Resources	• Skillful and experienced professionals [32, 33, 47]; • Staff training and education [7, 27, 33, 34, 45]; • Employees’ engagement [27, 29]; • Agile teams [46]	• Lack of skillful and experienced professionals [32, 33]
	Marketing	• Market sensitivity and customer focus [29]	/
	Operation management	• Flexible resource capacity [29]; • Possibility to quickly reconfigure production plan and processes [47]; • Product design by consumer demand [47]; • Short life cycles for timely delivery [29, 47]; • Sufficient use of shared resources [22, 40] • Speed in improving consumer service and delivery reliability [47]; • Cold chain technology [31]; • Standardization [7, 27, 34, 45]; • Modularization and division of processes [7, 33, 34, 42, 44, 45, 47]; • Well defined decoupling points [40]; • Design for manufacturing [47]	• Difficulty to control outsourced processes and performance monitoring problem as a risk management issue [26]
	Technology	• Information technology [46, 47]	• Risks concerning end-users, vendors, systems and infrastructure [35]

## Discussion

### Theoretical contribution

There is no evidence showing the existence of systematic literature review on lean and agile operation in healthcare management. Dixit and his colleagues [1] conducted a systematic literature review of healthcare supply chain (HSC), but the review just mentioned lean and agile operation as one of the many important aspects of the area. This study fills the gap by providing consolidated knowledge on the topic, allowing more in-depth understanding of the theory and explore potential gaps for future research.

By presenting different perceptions of leagility in the healthcare sector, this study reveals the evolution of the concept across researched timeframe and indicates how it could fit into the healthcare context. As empirical validation of leagility is still scarce, consolidating its manifold perceptions and interpretation in the healthcare sector is vital to construct a more holistic conceptual framework for leagile healthcare. This allows in-depth understanding of the concept and thus better guides leagility implementation in the healthcare context. And vice versa, the practice of leagile healthcare provides more evidence on its potential benefits to the healthcare system.

### Practical contribution

The contribution to practice of this study is threefold.

First, 11 target areas in current study are listed, including hospital-wide [26, 27, 29, 40, 42, 44, 46], overall health system [1, 30], patient flow [7, 22, 28, 32–34], pharmacy [20, 35], equipments [35], clinical laboratory [21], healthcare technology [36], point-of-care (POC) diagnosis [43], communication process [41], operating room (OR) cleaning [45] and vaccine supply chain [31]. This provides guidance for practitioners to apply leagility theory in respective sectors. Moreover, it leaves a hint for future research to explore areas that has not been mentioned yet, such as intensive care unit (ICU), organ transplant centers, mental health units and other departments within a hospital. Nevertheless, primary healthcare, elderly care centers and many other services across the healthcare industry also await for further study.

Second, the applicability of leagility in healthcare area is identified [15, 20], which enables practitioners to make decisions whether and where the leagile strategy could be adopted to improve organizational efficiency and effectiveness. By adopting leagility principles at the right place, it is more likely to achieve best quality healthcare services at a controlled price [20].

Third, success and limiting factors of leagility application in healthcare are classified by macro and micro aspects and further categorized into six dimensions: policy, organizational, human resources, marketing, operation management and technology. At macro political level, it is essential to design refined financial regimes to ensure reseasonable financial support for making the best decision at the decoupling point [32]. In the case of Uslu and her colleagues [32], the difference in reimbursement of laparoscopic and open surgery led to the dilemma of choosing a clinical decision better for the patient or the organization. Imperfect financial regimes may cause unnecessary suffering to the patient even though the decision may bring more benefits to the healthcare organization. It is also implied that instead of considering merely organizational efficiency, the lean and agile approach should rather be patient oriented. In organizational aspects, it is most vital to gain leadership support to carry out a top-down leagile reform [21, 26, 27]. A system-wide strategy is essential for leagility to achieve greater influence throughout the organization [7, 26, 28]. In the dimension of human resources, as lack of skillful and experienced professionals constructs one of the constraints of successful implementation of leagile strategy [32, 33], training and education as well as better human resources management is indispensable to have and retain qualified labor force. Employee’s engagement refers to trust and empower instead of control and going over into details [29]. An agile team is highly autonomous [46], thus high level surveillance and perfectionism from supervisors might need to be avoided during execution of the strategy. From the angle of operation management, standardization and modularization of processes gain the highest rate of exposure beyond any other factors. Modularization was observed in managing patient flow [7, 27, 33, 34], material logistics [44] and pharmacy [47]. Standardization was found to be utilized in patient treatment process [7, 34], operating room cleaning [45] and staff training [27]. Both of them serve to streamline processes and improve efficiency of the system. Additionally, monitor and risk management is required for outsourcing activities [26]. Concerning technological issues of leagility implementation, it is also mentioned by researchers to consider the risk related to end-users and vendors, such as privacy problems when adopting a new technology [35]. This categorization helps practitioners identify what to promote and reinforce in field work as well as what should be averted for a positive outcome while implementing leagility in the healthcare sector.

### Research gaps and indication for future study

In general, leagility in healthcare settings is a rather “young” concept that awaits for further development [1, 7, 29]. Due to its being in an early stage, limited cooperation between different authors was observed in current studies. Thus more collaboration among scholars is appealed for further exploration on this concept, as well as healthcare situations that are available to benefit from its potential.

Although there is a consecutive growth of published articles concerning leagile healthcare, most of them are theoretical and lack empirical validation. To fill this gap, first-hand data collected from real field could be used to analyze the applicability of leagility in healthcare and how the concept affects performance of the system. Additionally, proposed models and conceptual framework in existing knowledge could be applied in healthcare organizations of different levels and scales [7, 42], identifying and exploring the healthcare scenarios and cases that require specific adjustments.

Over 50% of the presented studies were conducted in Europe while the remaining half are distributed sparsely in other countries. Research is not yet spotted in many countries or regions such as China, Australia, Africa and South America. Several of these regions and some parts of these countries are not very advanced in terms of their healthcare offerings and could benefit from a more structured service if leagile principles are considered. This indicates a geographical gap to be filled in future studies, allowing the identification of eventual regional or system structural particularities in the adoption of the leagile principle.

Moreover, scholars mostly hold a system-wide view towards leagile healthcare or focus mainly on patient flow in healthcare services. Application of leagility in a specific sub-sector under the healthcare setting appears only after 2015 [20, 26, 28, 36, 45–47]. It is worth exploring the adoption of leagility in various sectors across a healthcare organization, such as research and clinical trials, medical education and training management and surgical operation management to improve performance in more areas under healthcare settings.

### Limitation

The fact that non-English written articles are not included in the study might pose as a limitation to the study since it may lead to bias or miss of information on the concept. Nonetheless, Chinese language was considered, and with it a potential wide range of articles, as the Chinese Government is focusing heavily on the reorganization of its system [48–50]. Additionally, this research constructed points of view based merely on ideas or results presented by other scholars without considering the views of filed practitioners or incorporating primary data. But with such early development of the topic and the fact that the research aimed at performing a systematic literature review, such inclusion would not have been appropriate.

## Conclusion

Leagility in the healthcare context is still being in its infancy with potential to improve healthcare services. To the best of our knowledge, this is the first systematic literature review consolidating knowledge on leagile healthcare. The 11 target areas of leagility application in the healthcare sector include hospital-wide implementation, patient flow, overall healthcare system, pharmacy, equipments, clinical laboratory, healthcare technology, point-of-care (POC) diagnostics, communication process, operating room (OR) cleaning and vaccine supply chain. Many healthcare areas are still unexplored and call for empirical validation of benefits brought from leagility. Moreover, success and limiting factors of leagility in healthcare were classified by macro and micro aspects and further categorized into six dimensions: policy, organization, human resources, marketing, operation management and technology. A majority of influencing factors fall within the category of organization and operation management. Standardization and modularization are the two most frequently mentioned factors for a successful leagility application in healthcare. Besides, leadership support, a system-wide strategy, better planning and control and skillfulness of employees are also vital elements to consider while adopting the leagility approach. This finding helps field practitioners better understand what should be facilitated or averted when using leagility to improve the performance of a healthcare system. Lastly, Four research gaps are identified and indication for future research is proposed.

### Supplementary Information

**Supplementary Material 1.**

## Data Availability

No datasets were generated or analysed during the current study.
