# Supplementary material for: Leagility in the healthcare research: a systematic review
Source: BMC Health Serv Res. 2024 Mar 7;24:307. doi: 10.1186/s12913-024-10771-0 (PMC10921567; doi:10.1186/s12913-024-10771-0)
Supplement: Supplementary file 1 — Supplementary Material 1. [file 12913_2024_10771_MOESM1_ESM.zip › Annex 1.docx]

Annex 1

Quality appraisal report of included studies

| Studies | *1* | *2* | *3* | *4* | *5* | *6* | *7* | *8* | *9* | *10* | *11* | *T* |  |
| --- | --- | --- | --- | --- | --- | --- | --- | --- | --- | --- | --- | --- | --- |
| 1. Vries et al (42) | 1 | 1 | 1 | 1 | 1 | 0.5 | 1 | 0.5 | 1 | 1 | 1 | 10 |  |
| 2. Toussaint et al (47) | 1 | 1 | 1 | 1 | 1 | 1 | 0.5 | 0.5 | 0.5 | 1 | 0.5 | 9 |  |
| 3. Towill and Christopher (46) | 1 | 1 | 1 | 1 | 0.5 | 1 | 0.5 | 1 | 1 | 1 | 1 | 10 |  |
| 4. Rahimnia and Moghadasian (33) | 1 | 1 | 1 | 1 | 1 | 1 | 1 | 1 | 1 | 1 | 1 | 11 |  |
| 5. Aronsson et al (7) | 1 | 1 | 1 | 1 | 1 | 1 | 1 | 1 | 1 | 1 | 1 | 11 |  |
| 6. Saghafian et al (22) | 1 | 1 | 1 | 1 | 1 | 1 | 1 | 1 | 1 | 1 | 1 | 11 |  |
| 7. Guimarães and Carvalho (27) | 1 | 1 | 1 | 1 | 1 | 1 | 0.5 | 0.5 | 1 | 1 | 1 | 10 |  |
| 8. Guimarães and Carvalho (26) | 1 | 1 | 1 | 1 | 1 | 1 | 0.5 | 0.5 | 0.5 | 0.5 | 0.5 | 8.5 |  |
| 9. Uslu et al (32) | 1 | 1 | 1 | 1 | 1 | 1 | 1 | 1 | 1 | 1 | 1 | 11 |  |
| 10. Olsson and Aronsson (28) | 1 | 1 | 1 | 1 | 1 | 1 | 1 | 0.5 | 1 | 1 | 1 | 10.5 |  |
| 11. Tolf et al (43) | 0.5 | 1 | 0.5 | 1 | 1 | 1 | 0.5 | 0.5 | 0.5 | 0.5 | 0.5 | 7.5 |  |
| 12. Pérez et al (21) | 1 | 1 | 1 | 1 | 1 | 1 | 0.5 | 0.5 | 1 | 1 | 0.5 | 9.5 |  |
| 13. Kuupiel et al (40) | 0.5 | 1 | 0.5 | 1 | 1 | 1 | 0.5 | 0.5 | 1 | 0.5 | 0.5 | 8 |  |
| 14. Nabelsi and Gagnon (35) | 1 | 1 | 1 | 1 | 1 | 1 | 1 | 1 | 1 | 1 | 1 | 11 |  |
| 15. Wikner et al (34) | 1 | 1 | 1 | 1 | 1 | 1 | 1 | 1 | 1 | 1 | 1 | 11 |  |
| 16. Dixit et al (1) | 1 | 1 | 0.5 | 1 | 1 | 1 | 1 | 0.5 | 0.5 | 0.5 | 0.5 | 8.5 |  |
| 17. Mishra et al (20) | 1 | 1 | 1 | 1 | 1 | 1 | 1 | 0.5 | 0.5 | 0.5 | 0.5 | 9 |  |
| 18. Pohjosenperä et al (45) | 1 | 1 | 1 | 1 | 1 | 1 | 1 | 1 | 1 | 1 | 1 | 11 |  |
| 19. Ni et al (36) | 0.5 | 1 | 1 | 1 | 1 | 1 | 0.5 | 0.5 | 0.5 | 0.5 | 0.5 | 8 |  |
| 20. Claudio et al (44) | 1 | 1 | 1 | 1 | 1 | 1 | 0.5 | 0.5 | 0.5 | 0.5 | 0.5 | 8.5 |  |
| 21. Sen et al (30) | 0.5 | 0.5 | 0.5 | 0.5 | 0.5 | 0.5 | 0.5 | 0.5 | 0.5 | 0.5 | 0.5 | 5.5 |  |
| 22. Al Fannah et al (48) | 1 | 1 | 1 | 1 | 1 | 1 | 0.5 | 0.5 | 1 | 0 | 0 | 8 |  |
| 23. Yadav and Kumar (31) | 1 | 1 | 1 | 1 | 1 | 1 | 1 | 1 | 1 | 0.5 | 1 | 10.5 |  |
| 24. Saraji et al (41) | 1 | 1 | 1 | 1 | 1 | 1 | 1 | 1 | 1 | 0.5 | 1 | 10.5 |  |
| Note: 1. Does the literature review justify the study 2. Is there a clear study aim 3. Do the study clearly define outcome, and other variables that need to be measured 4. Is the sample recruitment process clear 5. Are sample characteristics adequately described 6. Has sample size been justified 7. Are the methods described clearly and has been validated 8. Methods of assessing outcome valid (sensitive and specific, accurate and precise) 9. Can you follow the analysis and the tables and text are clear 10. Is the discussion fair and balanced and covered the strengths and weaknesses of their study 11. Are conclusions justified and appropriate to the results as presented | | | | | | | | | | | | | |
